# Association between Adverse Maternal Clinical Outcomes and Imbalance of Cytokines and Angiogenic Factors in Preterm Preeclampsia

**DOI:** 10.1055/s-0041-1735157

**Published:** 2021-10-20

**Authors:** Priscila Rezeck Nunes, Mariana Romao-Veiga, Vera Therezinha Medeiros Borges, Mariana Leticia Matias, Vanessa Rocha Ribeiro, Roberto Antonio Araujo Costa, Maria Terezinha Serrao Peracoli, Jose Carlos Peracoli

**Affiliations:** 1 Faculdade de Medicina, Universidade Estadual Paulista Julio de Mesquita Filho, Botucatu, SP, Brazil

**Keywords:** angiogenic factors, clinical adverse outcomes, cytokines, preterm preeclampsia, term preeclampsia, fatores angiogênicos, desfechos clínicos adversos, citocinas, pré-eclâmpsia pré-termo, pré-eclâmpsia a termo

## Abstract

**Objective** Preeclampsia (PE) is a pregnancy-specific syndrome characterized by abnormal levels of cytokines and angiogenic factors, playing a role in the disease development. The present study evaluated whether immunological markers are associated with the gestational age and with the disease severity in preeclamptic women.

**Methods** Ninety-five women who developed PE were stratified for gestational age as preterm PE (< 37 weeks) and term PE (≥ 37 weeks of gestation) and compared for disease severity as well as plasma concentration of angiogenic factors and cytokines. The concentrations of placental growth factor (PlGF), vascular endothelial growth factor (VEGF), Fms-like soluble tyrosine kinase (sFlt-1) and soluble endoglin (sEng), as well as the cytokines, tumor necrosis factor-α (TNF-α) and interleukin 10 (IL-10), were determined by enzyme-linked immunosorbent assay (ELISA).

**Results** The comparison between preeclamptic groups showed a higher percentage of severe cases in preterm PE (82.1%) than in term PE (35.9%). Similarly, the concentrations of TNF-α, sFlt-1, and sEng, as well as TNF-α/IL-10 and sFlt-1/PlGF ratios were significantly higher in the preterm PE group. In contrast, concentrations of PlGF, VEGF, and IL-10 were significantly lower in women with preterm PE. Negative correlations between TNF-α and IL-10 (r = 0.5232) and between PlGF and sFlt1 (r = −0.4158) were detected in the preterm PE.

**Conclusion** In pregnant women with preterm PE, there is an imbalance between immunological markers, with the predominance of anti-angiogenic factors and TNF-α, associated with adverse maternal clinical outcomes.

## Resumo

**Objetivo** A pré-eclâmpsia (PE) é uma síndrome específica da gravidez caracterizada por níveis anormais de citocinas e fatores angiogênicos, que desempenham um papel no desenvolvimento da doença. Este estudo avaliou se os marcadores imunológicos estão associados à idade gestacional e à gravidade da doença em mulheres com pré-eclâmpsia.

**Métodos** Noventa e cinco mulheres que desenvolveram PE foram estratificadas pela idade gestacional em PE pré-termo (< 37 semanas) e PE a termo (≥ 37 semanas de gestação) e comparadas quanto à gravidade da doença, bem como à concentração plasmática de fatores angiogênicos e citocinas. As concentrações de fator de crescimento placentário (PlGF), fator de crescimento endotelial vascular (VEGF), tirosina quinase solúvel semelhante a Fms (sFlt-1) e endoglina solúvel (sEng), bem como as citocinas, fator de necrose tumoral alfa (TNF- α) e interleucina 10 (IL-10), foram determinados porensaio de imunoabsorção enzimática (ELISA, na sigla em inglês).

**Resultados** A comparação entre os grupos com pré-eclâmpsia mostrou maior porcentagem de casos graves em PE pré-termo (82,1%) do que em PE a termo (35,9%). Da mesma forma, as concentrações de TNF-α, sFlt-1 e sEng, bem como as razões TNF-α/IL-10 e sFlt-1/PlGF foram significativamente maiores no grupo de PE pré-termo. Em contraste, as concentrações de PlGF, VEGF e IL-10 foram significativamente menores em mulheres com PE pré-termo. Correlações negativas entre TNF-α e IL-10 (r = 0.5232) e entre PlGF e sFlt1 (r = −0.4158) foram detectadas no grupo de PE pré-termo.

**Conclusão** Em gestantes com PE pré-termo, ocorre um desequilíbrio entre os marcadores imunológicos, com predomínio de fatores antiangiogênicos e TNF-α, associados a desfechos clínicos maternos adversos.

## Introduction

Pregnancies complicated by preeclampsia (PE), a specific human multisystemic syndrome, are the major cause of maternal and fetal morbidity and mortality worldwide. 1 2 Preeclampsia diagnosis is made through the identification of hypertension and proteinuria from 20 weeks of gestation. More recently, in the absence of proteinuria, the presence of target organ damage, such as maternal neurological or hematological complications; renal insufficiency; and impaired liver function is also considered as preeclampsia. 1 3 4

Some published data suggest that PE may be better understood if segregated into distinct phenotypes, with different etiologies and clinical manifestations. 5 6 7 This phenotypic classification may be based on the gestational age at time of disease diagnosis. Preterm PE (identified before 37 weeks) commonly represents a more severe and complicated type of PE than when this disease occurs at term (identified from 37 weeks of gestation). 8 9

The etiology of PE remains to be appropriately elucidated, despite the many studies already performed. The widely accepted abnormalities in the pathophysiology of PE include the involvement of angiogenesis, oxidative stress, and inflammation. 10 11 Thus, PE is characterized by the detection of abnormal levels of cytokines and angiogenic factors in the placenta and maternal circulation, suggesting that these immunological factors play a role in disease development. 12 13 14 15

Previous studies by our research group have shown elevated inflammatory cytokine levels in plasma from preeclamptic women, with higher levels of interleukin-1 β (IL-1β), IL-12, and tumor necrosis factor-α (TNF-α) associated with disease severity. 13 It is already well established that preeclamptic patients present excessive production of proinflammatory cytokines as well as deficient production of interleukin-10 (IL-10), a regulatory cytokine, in the placenta and maternal circulation. 16 17 18 19

The imbalance in circulating proangiogenic and antiangiogenic factors in the maternal circulation of preeclamptic women showed that the soluble form of the protein fms-like tyrosine kinase-1 (sFlt-1) exerts antiangiogenic effects by binding and inhibiting the biological activity of the proangiogenic proteins vascular endothelial growth factor (VEGF) and placental growth factor (PlGF). 20 21 Soluble endoglin (sEng) is another antiangiogenic protein that is detected at high levels in the plasma of preeclamptic women, and acts as a co-receptor for transforming growth factor β (TGF-β), hindering the role of this cytokine in maintaining a healthy endothelium. 12 22 23

In preterm PE, there is an increase in sFlt-1 and sEng serum levels followed by decreased levels of PlGF. 12 According to the literature, an imbalance between these proangiogenic and antiangiogenic proteins in the plasma of preeclamptic women was correlated with disease severity and may have the ability to predict adverse maternal or fetal outcomes and contribute to the pathogenesis of PE. 12 21 24

Thus, given previous reports suggesting that preterm PE is phenotypically different from PE occurring at term, we hypothesized that there are differences in the concentrations of cytokines and the angiogenic factors in the plasma of these women. The present study, therefore, aimed to determine whether an imbalance of immunological markers is associated with gestational age and disease severity at the time of diagnosis of the disease.

## Methods

### Subjects

This was a prospective, cross-sectional, observational study of 95 pregnant women without a previous history of hypertension or obstetric and medical complications, who were diagnosed with PE according to the American College of Obstetricians and Gynecologists (ACOG) 1 and defined as the onset of persistently elevated blood pressure of 140/90 mm Hg, associated or not with proteinuria (≥ 300 mg in urine collected during 24 hour) and other severe clinical complications after the 20th week of gestation. These 95 patients were admitted to the obstetric unit of Faculdade de Medicina de Botucatu, Botucatu, SP, between March 2017 and July 2018. Gestational age was calculated from the last menstrual period and confirmed by early ultrasound examination (< 14 weeks gestation). The pregnant women were stratified for gestational age as preterm PE (< 37 weeks) and term PE (≥ 37 weeks of gestation). Preeclampsia with severe features was diagnosed according to the ACOG, 1 including the following parameters: systolic blood pressure ≥ 160 mm Hg or diastolic blood pressure ≥ 110 mm Hg; proteinuria ≥ 2,000 mg/24h; new-onset cerebral or visual disturbance, such as photopsia (flashes of light) and/or scotomata (dark areas or gaps in the visual field); severe headache or headache that persists and progresses despite analgesic therapy; altered mental status; eclampsia; severe, persistent right upper quadrant or epigastric pain; serum transaminase concentration ≥ 2 times the upper limit of normal; < 100,000 platelets/microL; progressive renal insufficiency (serum creatinine > 1.1 mg/dL or 97.3 micromol/L); hemolysis, elevated liver enzymes, low platelet count (HELLP) syndrome and pulmonary edema. Proteinuria in 24-hour urine was measured by a colorimetric method, the Technicon RA-X automation system (Vetra-Tech Services, Houston, USA) in the clinical laboratory Faculdade de Medicina de Botucatu–Universidade Estadual Paulista (UNESP).

### Exclusion Criteria and Ethical Approval

The exclusion criteria included multiple gestations, illicit drug use, and preexisting medical conditions, such as diabetes, chronic hypertension, infections, autoimmunity, hepatic, and renal disease. The ethics committee of Faculdade de Medicina de Botucatu approved the study (protocol number 3923–2011), and all women signed the written informed consent. Parents or guardians signed for women younger than 18 years old. All experiments reported in this paper were performed under the health and safety procedures and following relevant guidelines and regulations from the Code of Ethics of the World Medical Association (Declaration of Helsinki).

### Blood Sampling

Peripheral blood samples (10 mL) were collected from preeclamptic women at the time of disease diagnosis by venipuncture from the antecubital vein and were put into a sterile plastic tube that contained 10 U/ml ethylenediaminetetraacetic acid (EDTA; Becton Dickinson-BD Vacutainer; BD Biosciences, Franklin Lakes, NJ, USA). After blood centrifugation at 4°C for 10 minutes at 1,200 g, the plasma fraction was removed, and aliquots were stored at -80° for angiogenic factors and cytokines determination.

### Determination of Cytokines and Angiogenic Factors

The cytokine concentrations and angiogenic factors in plasma were determined shortly thereafter, and blood collection was performed with Quantikine enzyme linked immunosorbent assay (ELISA) kits (R&D Systems, Minneapolis, MN, USA) for TNF-α, IL-10, PlGF, VEGF, sFlt-1 and endoglin, according to the manufacturer's instructions. Assay sensitivity limits were 6.23 pg/mL for TNF-α, 3.9 pg/mL for IL-10, 7.0 pg/mL for PlGF, 9.0 pg/mL for VEGF, 8.46 pg/mL for sFLT-1, and 0.03 ng/mL for endoglin.

### Statistical Analysis

The clinical characteristics of preeclamptic women, as well as cytokines determination and angiogenic factors, were analyzed by non-parametric methods (Mann-Whitney U test and Chi-squared test). The results were evaluated using the statistical program GraphPad Prism, version 6.01 (GraphPad, La Jolla, CA, USA), and statistical significance was accepted at *p* < 0.05.

## Results

### Clinical Features

The clinical characteristics of women with PE are shown in Table 1 . Of the 95 women with PE, 56 (58.9%) had a preterm birth (< 37 w), and 39 (41.1%) had a term birth (≥ 37w). No significant differences in the relation of age were detected between the groups studied. The comparison between preterm and term PE showed significant differences in gestational age. Higher levels of systolic and diastolic blood pressure as well as proteinuria were detected in the preterm PE group. The percentage of severe cases with target organ damage was higher in the preterm PE (82.1%) than in the term PE (35.9%) group.

**Table 1:** Clinical features of women with preeclampsia

Parameters	Preterm preeclampsia (< 37 weeks of gestation) n = 56	Term preeclampsia (≥ 37 weeks of gestation) n = 39	*p* -value
Age (years)	25 (15–40)	23 (16–37)	NS
Gestational age (weeks)	33 (24–36)	38 (37–41)	< 0.001 *
Systolic blood pressure (mmHg)	160 (140–180)	140 (140–170)	0.0031 *
Diastolic blood pressure (mmHg)	110 (90–120)	100 (90–110)	0.0002 *
Proteinuria	2,550 (300–22,520)	515 (300–11,780)	< 0.0001 *
Target organ damage			
Absent	10 (17.9%)	25 (64.1%)	< 0.001 **
Present	46 (82.1%)	14 (35.9%)	< 0.001 **

Results are expressed as median (range min - max).

* Mann-Whitney U-test.

** Chi-squared test.

The occurrence of severity signals in preterm and term PE is described in Table 2 . The more frequent severe signals were observed in preterm PE compared with term PE and were represented by hypertensive crisis and imminent eclampsia. The percentage of adverse outcomes within the preterm PE group, with gestational age from 24 to 33 weeks, was 60.0% and is similar to that of the preterm group, with gestational age between 34 and 36 weeks and 6 days of gestation, with 71.4% of adverse outcomes. Absence of severity was detected in a higher percentage of patients with term PE (79.5%) while the association of two or more severe signals was more frequent in women with preterm PE.

**Table 2:** Preeclamptic women distribution according to absence or presence of severity signals and preterm or term preeclampsia

Variables	Preterm preeclampsia (< 37 weeks of gestation) n = 56 n (%)	Term preeclampsia (≥ 37 weeks of gestation) n = 39 n (%)
Hypertensive crisis	20 (35.7)*	3 (7.7)
Imminent eclampsia	15 (26.8)*	7 (17.9)
Eclampsia	3 (5.4)	0 (0)
Partial HELLP	8 (14.3)	2 (5.1)
HELLP	1 (1.8)	0 (0)
Pulmonary edema	2 (3.6)	0 (0)
Absence of severity	16 (28.6)	31 (79.5)*
Two or more severe signals	10 (17.9)*	1 (2.6)

Abbreviation: HELLP, hemolysis, elevated liver enzymes, low platelet count.

** *p* < 0.05 (Chi-squared test).

### Plasmatic Concentration of Cytokines in Preterm and Term PE

**Fig. 1:**
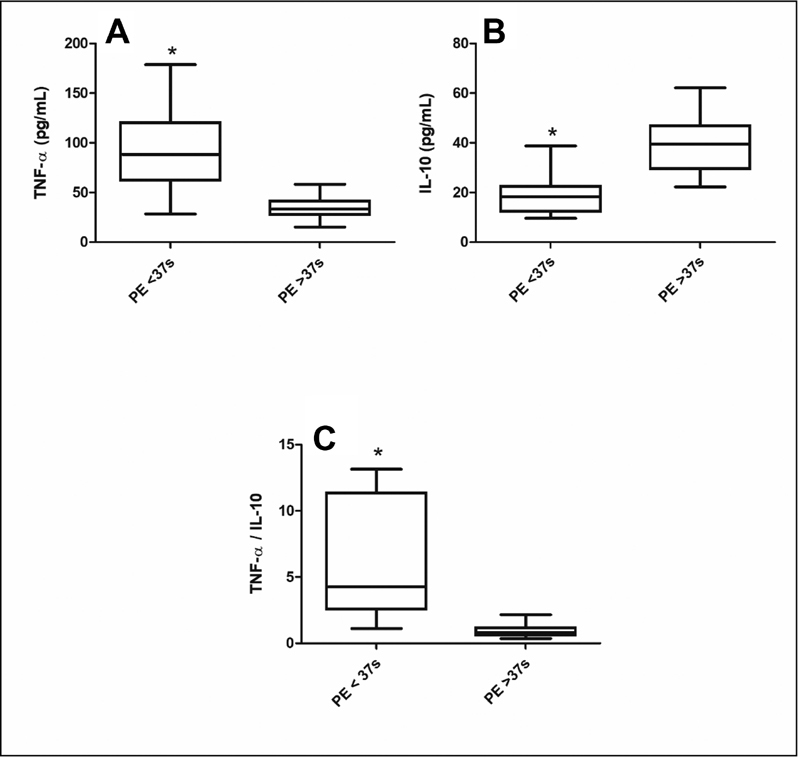
Plasmatic concentrations of TNF-α ( **A** ), IL-10 ( **B** ), and TNF-α/IL-10 ratio ( **C** ) in preterm and term PE. Results are expressed as median (horizontal line), 25 ^th^ and 75 ^th^ percentile (box), and range (whiskers). *( *p* < 0.001) vs PE ≥ 37 weeks (Mann-Whitney *U* test).

The concentration of TNF-α in plasma was significantly higher while IL-10 levels were significantly lower in the preterm preeclamptic group than in the term PE group. The TNF-α/IL-10 ratio showed a statistically significant increase in women with preterm PE ( Fig. 1 ). A negative correlation between TNF-α and IL-10 (r = - 0.5232; *p* < 0.005) was observed in the preterm PE group, whereas a non-significant correlation was detected in the group of term PE (r = - 0.2304; *p* = 0.3737).

### Plasmatic Concentrations of Pro- and Anti-Angiogenic Factors in Preeclamptic Women

**Fig. 2:**
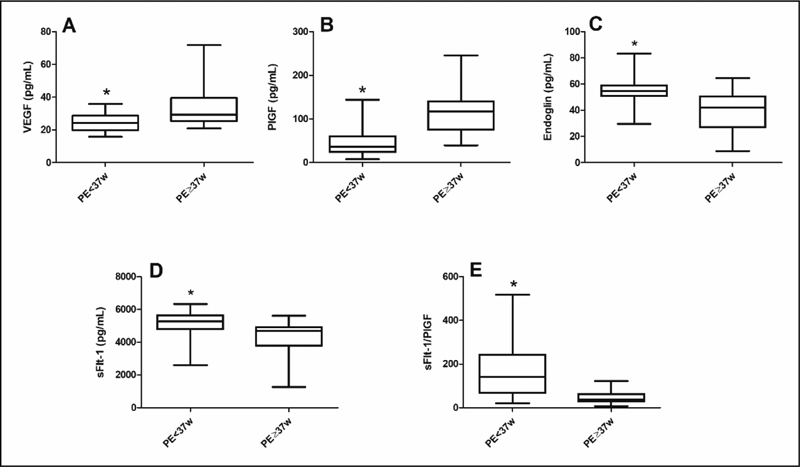
Plasmatic concentrations of the angiogenic factors VEGF ( **A** ) and PlGF ( **B** ), the anti-angiogenic factors endoglin ( **C** ), sFlt-1 ( **D** ), and sFlt-1/PlGF ratio (2E) in preterm (PE < 37 weeks of gestation) and term PE (> 37 weeks of gestation). Results are expressed as median (horizontal line), 25 ^th^ and 75 ^th^ percentile (box), and range (whiskers). *( *p* < 0.001) vs PE ≥ 37w (Mann-Whitney *U* test).

Fig. 2 shows that VEGF (2A) and PlGF (2B) plasma concentrations were significantly lower in the preterm PE group compared with the term PE group, while the anti-angiogenic factors Endoglin (2C) and sFlt-1 (2D) were significantly higher in preterm PE than in term PE group. The sFlt-1/PlGF ratio (2E) was increased in the preterm preeclamptic group compared with the term PE ones. Moreover, a significant negative correlation between sFlt1 and PlGF was detected in the preterm PE group (r = −0.4158; *p* < 0.005), whereas a non-significant correlation was observed in term PE group (r = -0.1887; *p* = 0.2930).

## Discussion

The main findings of the current study demonstrated that, compared with term PE, women with preterm PE showed an imbalance between immunological markers in plasma, with a predominance of higher levels of TNF-α and anti-angiogenic factors associated with adverse maternal clinical outcomes. Women who developed preterm PE had a higher concentration of proteinuria as well as higher systolic and diastolic blood pressure, and elevated frequency of target organ damage than women with term PE. The more frequent severe signals in women with preterm PE were hypertensive crisis and imminent eclampsia, followed by the appearance of partial HELLP syndrome and association of two or more severe signals. These results are in line with other authors, reporting that women with preterm PE show significantly higher levels of blood pressure, proteinuria, liver, and renal dysfunctions as well as neonatal morbidities. 9 On the other hand, the literature emphasizes that imbalance between immunological markers such as cytokines and angiogenic factors are more evident in early-onset preeclampsia (< 34 weeks) when compared with late-onset preeclampsia (≥ 34 weeks). 13 25 26 However, it is important to consider that the inclusion of term pregnancies in the late-onset PE group could minimize these alterations. Our present study suggests this possibility by showing that the imbalance of cytokines and angiogenic factors was significantly higher in preterm PE, including women with gestational age between 34 and 36 weeks and 6 days. These women, similarly to those with gestational age from 24 to 33 weeks, showed association between higher levels of immunological markers associated with a high percentage of maternal adverse outcomes, in comparison with the term PE group. Clinically, these results reflect the higher rate of adverse effects found in this preterm PE group and reinforces the need for greater care in the patient follow-up not only in early-onset PE, but also in preterm PE.

In the present study, the cytokine evaluation showed that the concentration of TNF-α in plasma was significantly higher, while IL-10 levels were significantly lower in the preterm PE group that showed association with more severe clinical signals compared with the term PE group. The imbalance between pro and antiinflammatory cytokines was represented by the TNF-α/IL-10 ratio, statistically significant increased, and a negative correlation between TNF-α and IL-10 (r = - 0.5232; *p* < 0.005) in the preterm PE group. These results agree with a previous study that showed the association of higher levels of the proinflammatory cytokines TNF-α, IL-1 β, and IL-12, as well as lower levels of IL-10 and disease severity in the early-onset PE group. 13 Several studies have demonstrated that proinflammatory cytokines are produced in excess by maternal immune cells in pregnancies complicated by preeclampsia, 16 18 27 and are responsible for the pathophysiological features, activating damage into the endothelial cells to initiate the maternal inflammatory responses. However, there are no studies evaluating the association of TNF-α and IL-10 with adverse outcomes in preterm and term PE. In opposition to the inflammatory cytokines, decreased levels of the regulatory cytokine IL-10, detected in the present study, confirm previous studies in preeclamptic women compared with normotensive pregnancies. 19 28 These findings highlight that IL-10 seems to not be able to exert its regulatory effects on proinflammatory cytokines, resulting in exacerbation of the systemic inflammatory response in PE.

The results of the present work showed that plasmatic concentrations of the proangiogenic factors VEGF and PlGF were significantly lower, while the antiangiogenic factors sEng and sFlt1 were significantly higher, causing an imbalance between angiogenic factors, revealed by an increase in sFlt1/PlGF ratio in the maternal plasma of women with preterm PE. This ratio in maternal circulation is usually employed as a routine for the prediction and/or diagnosis of PE. Our results are in line with studies showing that angiogenic imbalance is more prominent in preterm PE. 12 29 Elevated levels of circulating sFlt1 and decreased levels of PlGF are associated with adverse outcomes related to PE. 30 31 Fms-like soluble tyrosine kinase acts as an antagonist for VEGF and PlGF, removing free isoforms and avoiding their signaling in circulation. 32 Circulating sFlt-1 regulates excessive VEGF signaling in the maternal circulation during normal pregnancy. 33 In the same way, acting similarly to sFlt-1, sEng can contribute to maintaining endothelial function and vasculogenesis in the maternal circulation during pregnancy, through the binding to transforming growth factor-β. 34 However, high levels of sEng were detected in preeclamptic women with specific adverse outcomes, such as pulmonary edema, acute renal failure, cerebral hemorrhage, thrombocytopenia, elevated liver enzymes, and preterm delivery. Thus, circulating concentrations of angiogenic factors are intimately related to PE heterogeneity and are crucial to assess the severity of PE by their association with an increased risk of adverse outcomes. 35

The literature data showed that some authors have defended the importance of conducting clinical trials to observe whether risk stratification using angiogenic factors in patients with suspected PE can improve maternal and fetal results. Predictive and diagnostic tests for preterm PE and discussion on their clinical use and potential value in the management of preterm PE are extremely important in these cases. 31 35

## Conclusion

Together, the results of this study demonstrated that women with preterm PE have an imbalance between plasmatic levels of TNF-α and IL-10 and between PlGF and sFlt-1, associated with more adverse clinical outcomes.
